# Evaluating Michigan's community hospital access: spatial methods for decision support

**DOI:** 10.1186/1476-072X-5-42

**Published:** 2006-09-22

**Authors:** Joseph P Messina, Ashton M Shortridge, Richard E Groop, Pariwate Varnakovida, Mark J Finn

**Affiliations:** 1 Department of Geography, Michigan State University, East Lansing, MI, USA

## Abstract

**Background:**

Community hospital placement is dictated by a diverse set of geographical factors and historical contingency. In the summer of 2004, a multi-organizational committee headed by the State of Michigan's Department of Community Health approached the authors of this paper with questions about how spatial analyses might be employed to develop a revised community hospital approval procedure. Three objectives were set. First, the committee needed visualizations of both the spatial pattern of Michigan's population and its 139 community hospitals. Second, the committee required a clear, defensible assessment methodology to quantify access to existing hospitals statewide, taking into account factors such as distance to nearest hospital and road network density to estimate travel time. Third, the committee wanted to contrast the spatial distribution of existing community hospitals with a theoretical configuration that best met statewide demand. This paper presents our efforts to first describe the distribution of Michigan's current community hospital pattern and its people, and second, develop two models, access-based and demand-based, to identify areas with inadequate access to existing hospitals.

**Results:**

Using the product from the access-based model and contiguity and population criteria, two areas were identified as being "under-served." The lower area, located north/northeast of Detroit, contained the greater total land area and population of the two areas. The upper area was centered north of Grand Rapids. A demand-based model was applied to evaluate the existing facility arrangement by allocating daily bed demand in each ZIP code to the closest facility. We found 1,887 beds per day were demanded by ZIP centroids more than 16.1 kilometers from the nearest existing hospital. This represented 12.7% of the average statewide daily bed demand. If a 32.3 kilometer radius was employed, unmet demand dropped to 160 beds per day (1.1%).

**Conclusion:**

Both modeling approaches enable policymakers to identify under-served areas. Ultimately this paper is concerned with the intersection of spatial analysis and policymaking. Using the best scientific practice to identify locations of under-served populations based on many factors provides policymakers with a powerful tool for making good decisions.

## Background

**Figure 1:**
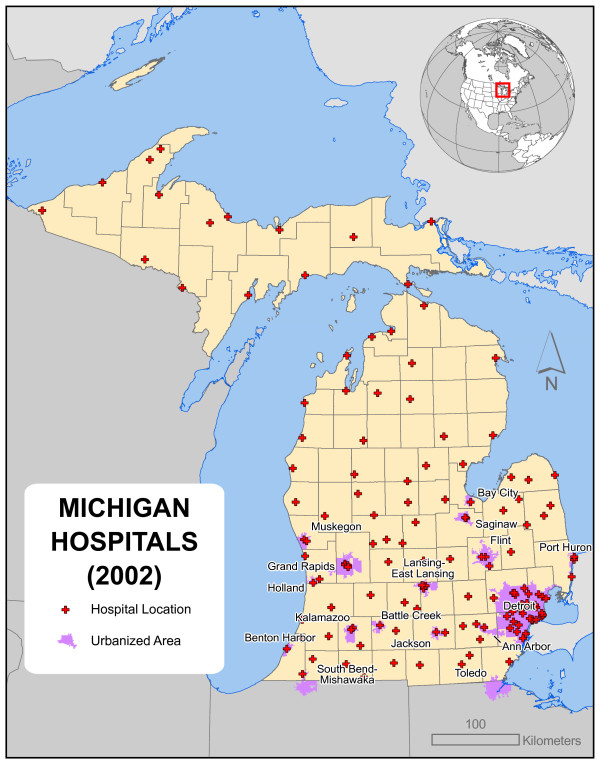
**The locations of Michigan's 139 community hospitals in 2002**. The factors leading to the development of today's spatial distribution of 139 community hospitals were largely local and particular for each individual hospital, and the current configuration emergent.

Community hospitals are situated where they are for many reasons. Some facilities were built to serve large local populations; others were intended to provide regional coverage across less populated areas. The precise settings of these hospitals were dictated by diverse factors of geographical and historical contingency, including the population distribution at the time each facility was constructed, the physical characteristics of available sites, and significantly, the human and political context of the moment. In the state of Michigan – the setting of the present work – it seems quite likely that the factors leading to the development of today's spatial distribution of 139 community hospitals were largely local, often political, and particular for each individual hospital. Figure 1 presents the resulting pattern.

In the summer of 2004, a multi-organization Certificate of Need committee headed by the State of Michigan's Department of Community Health approached the authors of this paper with questions about how spatial analyses might be employed to develop a revised community hospital approval procedure. Recall, that the Hospital Certificate of Need regulation (CON) was created in the late 1950s as a regulatory response to rising health costs believed caused by government control of hospital construction and licensing. However, the CON process was not actually designed to control rising health care costs. It was hoped regional planning with community involvement would educate the public to the increasing quality and expense of good hospital care and lead to public acceptance of rising costs. Michigan's version of the CON includes academics to provide scientific assistance to committee members. In this recent CON, policymakers wanted to contrast the impact of a proposed facility with statewide needs in a quantitative geographical context. In particular, the State was concerned about identifying populations with lengthy access times to existing community hospitals. Serving these populations would become a core element in the new facility proposal assessment. As with many modern geographical planning exercises, the committee hoped geographic information system (GIS) based approaches might provide powerful perspective on the problem [1-4].

Discussion with Michigan Department of Community Health personnel and other interested groups via the Certificate of Need policy mechanism resulted in three main objectives that comprise the topics of this paper. First, policymakers needed visualizations of both the spatial pattern of Michigan's population and its 139 community hospitals. Current population distribution was only one element, as population density changes over time. Further, identification of sub-population concentrations utilizing community hospital resources, were another key mappable factor. Second, the committee required a clear, defensible assessment methodology that was generalizable and used existing technology. The method would quantify access to existing hospitals statewide, taking into account factors such as distance to nearest hospital and road network density to estimate travel time. Travel times based on average representative speeds due to varying road types would also be applied [5]. Areas falling below a particular time threshold would be identified as inaccessible. The identified inaccessible areas were then employed as a criterion in the evaluation of new community hospital proposals. Third, the committee wanted to contrast the spatial distribution of existing community hospitals with a theoretical configuration that best met statewide demand. In contrast to the second objective, this objective employed patient days data to quantify the spatial pattern of recent demand, but did not consider access as a function of the road network. The results may identify the degree of sub-optimality of the existing pattern of hospitals. Perhaps more importantly, it could foster dialogue on the definition of an optimal hospital configuration, and of what spatial characteristics were and remain most important for Michigan's hospital system.

Taken together, the second and third objectives provided a valuable comparative opportunity to identify regions in the state most in need of a new community hospital. Since the methods employed entirely different models, contrasting results help policymakers understand some of the spatial complexity of both the demand and the accessibility dimensions of the problem. Two additional factors influenced both researchers and the committee: first, the deadline was, by law, 6 months from the initial meeting, and second, model formulation and parameterization was an evolving process throughout the time period. As the committee's understanding of geographic models improved, members were able to provide more appropriate guidance to the researchers. However, the model endured multiple incarnations as political and economic interests influenced the methods, but ultimately, the results presented here were well matched to policymaker needs.

### Demographic patterns

Key to any statewide planning effort is understanding the geographic distribution of both facilities and the population [6]. Figure 1 depicts the 2002 locations of Michigan's 139 community hospitals. Most are located in the southern half of the Lower Peninsula and concentrated in urban centers. Michigan has an unusual boundary which holds significance for several modeling issues in this paper; the state's land mass occupies two peninsulas. The Upper Peninsula shares its southwestern boundary with a largely rural portion of Wisconsin, while the southern edge of the Lower Peninsula abuts relatively densely populated areas in Indiana and Ohio; numerous community hospitals are located in these states including many close to the border. Much of the state's border falls within the Great Lakes. The Great Lakes contain many islands, all of which must be managed for health services, and are relatively inaccessible, at least for hospital traffic.

**Figure 2:**
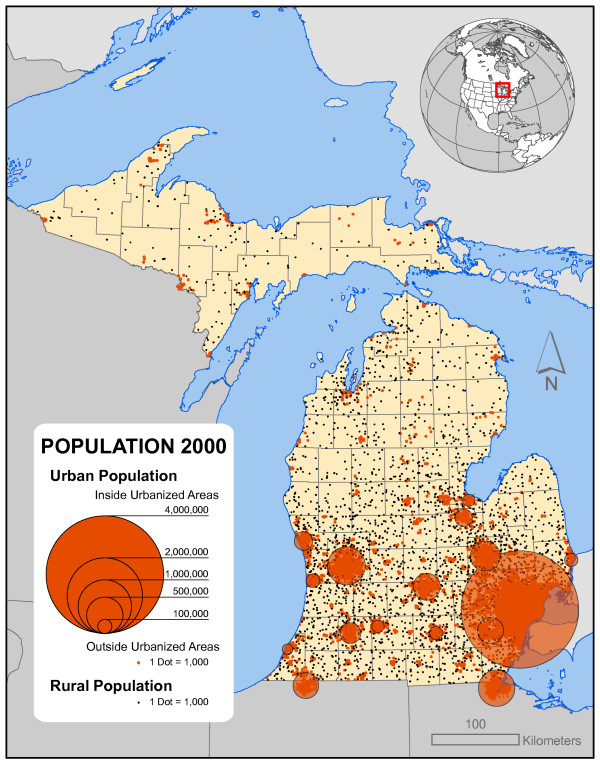
**Michigan urban and rural population distribution**. Most of Michigan's population is found south of the "Bay City Line" in the southern half of the Lower Peninsula with approximately 40% concentrated in the southeastern part of the state. North of that line, urban concentrations are few and rural population thins dramatically with the Upper Peninsula accounting for only 3.4% of the state's population.

**Figure 3:**
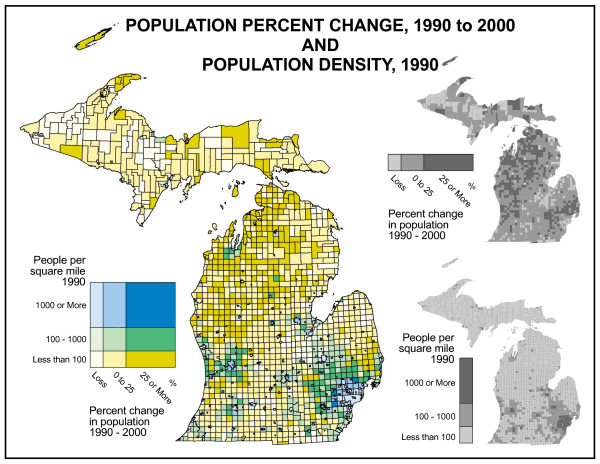
**Michigan population change and population density**. The highest population density area of the state is the Detroit metropolitan area with secondary areas around Grand Rapids and other cities. To the north, densities decline to some of the lowest in the eastern half of the U.S. Thus, Michigan provides one of the best examples of the highly varied nature of population distribution. Two distinct types of change are visible on this map. The first is the suburbanization of areas around the Detroit metro area, Grand Rapids, and other cities in the southern half of the Lower Peninsula where urban out-migrants are "sprawling" into the surrounding rural townships. The second type of migration is found in the northern half of the Lower Peninsula where urban and suburban migrants (mostly retirees) are locating in remote, rural locations in the quest for scenic amenities.

Figure 2 shows the state's population distribution. Most of Michigan's population is found south of the "Bay City Line" in the southern half of the Lower Peninsula with approximately 40% concentrated in the southeastern part of the state. North of that line, urban areas are few, and rural population thins dramatically with the Upper Peninsula accounting for only 3.4% of the state's population. Figure 3 depicts population change and population density across the state. The highest population density is found in the Detroit metropolitan area with secondary areas around Grand Rapids and other cities. To the north, densities decline to some of the lowest in the eastern half of the U.S. Thus, Michigan provides one of the best examples of the highly varied nature of population distribution. Of course this distribution is not static. Two distinct types of change are visible on this map. The first is the suburbanization of areas around the Detroit metro area, Grand Rapids, and other cities in the southern half of the Lower Peninsula where urban out-migrants are "sprawling" into the surrounding rural townships. The second is found in the northern half of the Lower Peninsula where urban and suburban migrants (mostly retirees) are locating in remote, rural locations for scenic amenities [7].

**Figure 4:**
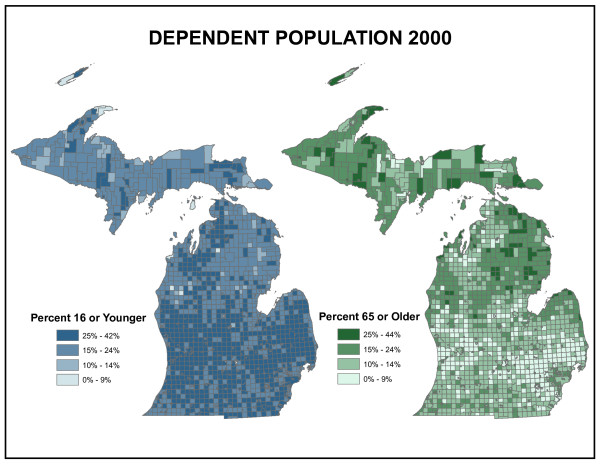
**Michigan dependent population**. These maps show the proportion of the population in Michigan under age 16 and over age 65 – the dependent population. Older people as a proportion of the population are concentrated in the northern half of the state, particularly in the Upper Peninsula and northeastern Lower Peninsula. These concentrations result both from the out-migration of younger people seeking employment or educational opportunities in large cities and the in-migration of older people seeking rural amenities.

**Figure 5:**
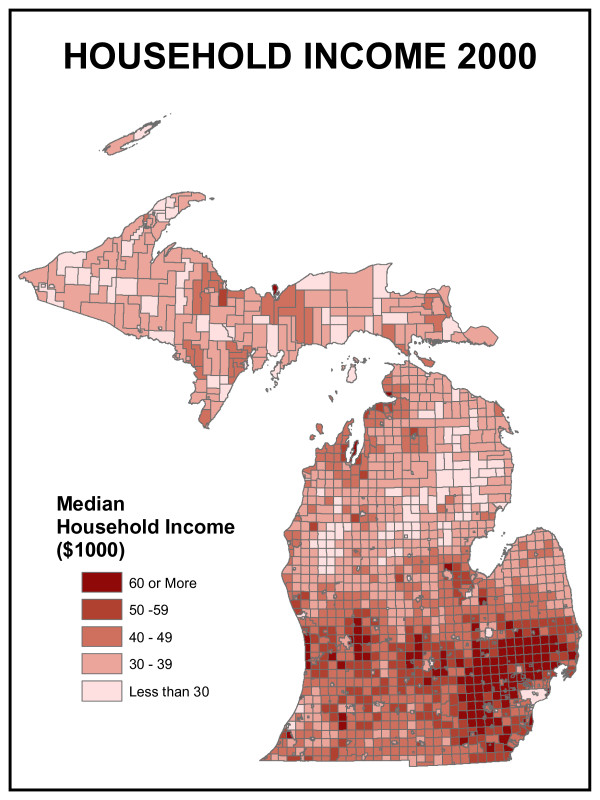
**Michigan household income**. Wealth, in the form of disposable income, is concentrated in the suburban areas of the southern half of the Lower Peninsula where income due to employment wages tend to be highest. Central cities such as Detroit, Flint and Lansing do not appear visually prominent on this map but are important "holes" in the distribution.

Hospital utilization is also a function of the age composition of the population. Figure 4 shows the proportion of the population in Michigan under age 16 and over age 65 – the dependent population. Older people as a proportion of the population are concentrated in the northern half of the state, particularly in the Upper Peninsula and northeastern Lower Peninsula. These concentrations result both from the out-migration of younger people seeking employment or educational opportunities in large cities and the in-migration of older people seeking rural amenities. Income is another traditional indicator of health care services demand. Figure 5 shows that wealth, in the form of disposable income, is concentrated in the suburban areas of the southern half of the Lower Peninsula where income due to employment wages tend to be highest. Central cities such as Detroit, Flint and Lansing do not appear visually prominent on this map but are important "holes" in the distribution.

### Access-based model of under-served areas

After providing a valuable overview of the statewide demographic patterns of importance to public health policy, the second research objective to address was concerned with the development of a travel time methodology to identify locations relatively remote from an existing community hospital. There have been many studies regarding travel times and accessibility to health services [5,8-10]. Some research has dealt with simple distance to nearest provider. Some has dealt with provider-to-population ratios [11]. Lauder et al. [12] stated that the previous work could be categorized into two types of analysis; first, modeling for traffic prediction, often associated with the Origin-Destination (O-D) networks, is mathematically intensive and requires extensive and often non-existent data, and second, modeling travel time for purposes where hospital accessibility is secondary. In addition, accessibility has been analyzed using Euclidian distances and Thiessen polygons [13,14]. Luo and Wang [15] examined spatial accessibility (SA) by using the Floating Catchment Area (FCA) method to define the service areas of physicians by a threshold travel time combined with a gravity-based model. Recently, researchers are beginning to combine the concepts of distance and supply under SA analysis [11]. The accessibility measure developed for this study is unique to the study but relies on well-accepted theoretical and computational foundations for support. While all the assumptions and model iterations are not presented in this document, the process of linking politics, policy, and model was quite involved.

#### Basic certificate of need requirements

1. 1 kilometer spatial resolution (reduced from 4.8 km originally)

2. All places in the state must be measured (includes islands and parks)

3. 30 minute travel time maximum to suitable hospitals [9,13]

4. Variations in road types must be considered (speed limits)

### Demand-based model of optimal facility location

The third research objective to address was to contrast the spatial distribution of existing community hospitals with a theoretical configuration that best met statewide demand. This theoretical configuration was generated using location-allocation modeling. Location-allocation modeling has been a significant geographic analytical tool for decades. Over that time, powerful models have been developed to identify optimal solutions for a variety of facility location and demand allocation problems [16-18]. Standard location models, regardless of form, require certain types of input information:

• Locations of existing facilities

• Locations of demand sources

• Locations representing potential sources

• A transportation network connecting these locations

The output of the location model is a set of new facility locations that optimally satisfy the demand, given assumptions and model constraints. These models define "optimal" in particular ways; for example, the P-median model identifies a solution that minimizes average (median) travel distance to the nearest facility from a set of demand locations. In its basic form, a P-median solution guarantees that aggregate travel is minimized. However, some demand points may be quite distant from the nearest facility. Public health applications may find other models more appropriate, especially since demand in this case is a person in (possibly urgent) need of medical care. The maximal covering location model (Maxcover) is an alternative that identifies facility locations so that as much demand as possible is within a specified distance of the closest facility [19]. More formally, this model maximizes the population covered within a specified distance of a specified number of facilities. The model solution is a set of facility locations that maximize coverage within a specified distance of those facilities. Maxcover-class problems have been employed in many health care problems, including emergency medical service location [20,21]. This paper employs the Maxcover-class solution.

## Results and discussion

### Under-served areas defined by access-based model

**Figure 6:**
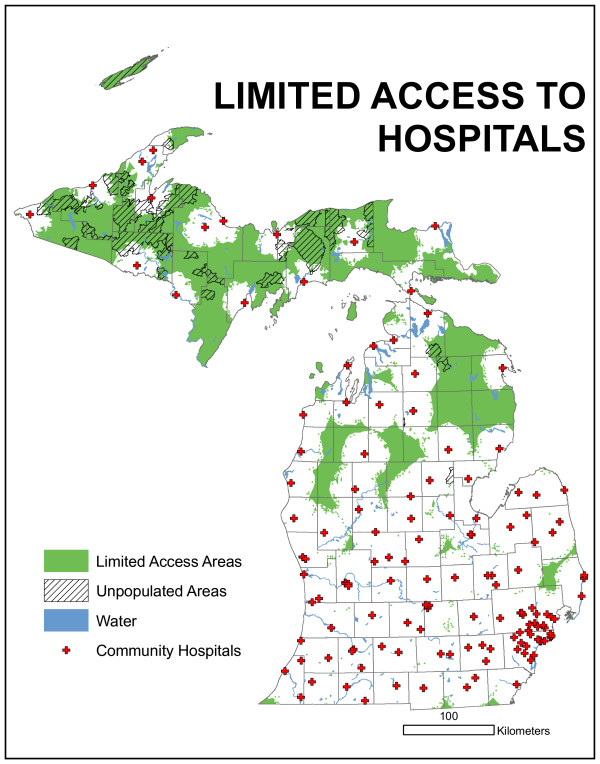
**Results of access-based model**. The Upper Peninsula contains the most area with poor medical access, but due to population totals and shifts, does not meet the criteria for an official under-served area. The northern Lower Peninsula also has a significant amount of area identified as poorly accessed, but also does not meet population criteria. There are three areas in the lower half of the Lower Peninsula that might meet the criteria: northeast of Detroit, north of Lansing, and north of Grand Rapids.

The access-oriented model adopted by the Certificate of Need committee as part of its formal facility proposal evaluation methodology is presented in map form in Figure 6. This map presents the results of the travel time methodology. Not surprisingly, the Upper Peninsula contains the greatest area with poor medical access, but due to population totals and shifts, it does not meet the criteria for an official under-served area. The northern Lower Peninsula also has a significant amount of area identified as poorly accessed, but also does not meet population criteria. There are three areas in the lower half of the Lower Peninsula that might meet the criteria: northeast of Detroit, north of Lansing, and north of Grand Rapids.

**Figure 7:**
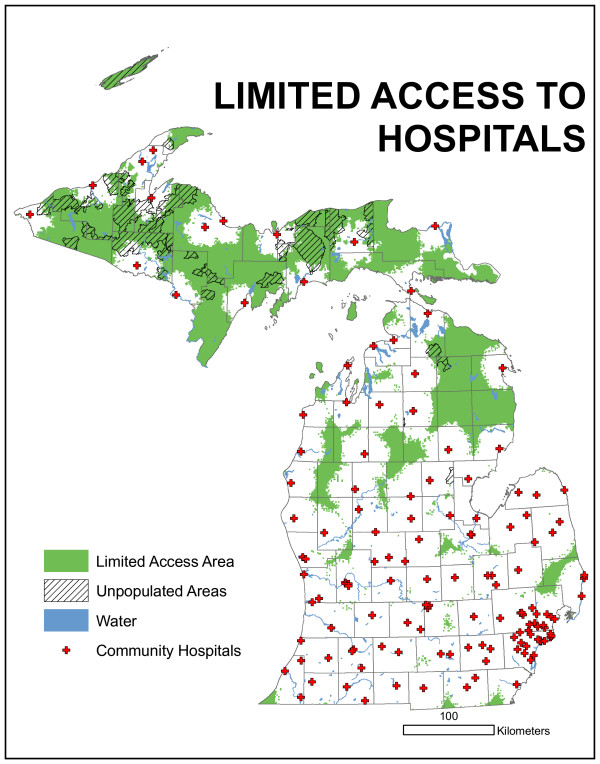
**Results of access-based model using a 25% urban road speed limit reduction**. The areas under-served essentially remain with slightly more total area now included. Careful comparison of Figure 6 with 7 permits the identification of new areas. However, this reduction in urban speed limits does not dramatically alter the configuration of the under-served areas.

One concern raised by the technical committee was with respect to rush hour travel times, specifically assuming travel delays. To address that concern, travel times were redefined in urban areas, i.e. urban functional classes, to account for a 25% reduction in speed limits. All other modeling parameters were held constant. This model output is presented in Figure 7. Using a 25% urban road speed limit reduction, the areas under-served essentially remain with slightly more total area now included. Careful comparison of Figure 6 with 7 permits the identification of new areas. However, this reduction in urban speed limits does not dramatically alter the configuration of the under-served areas.

**Figure 8:**
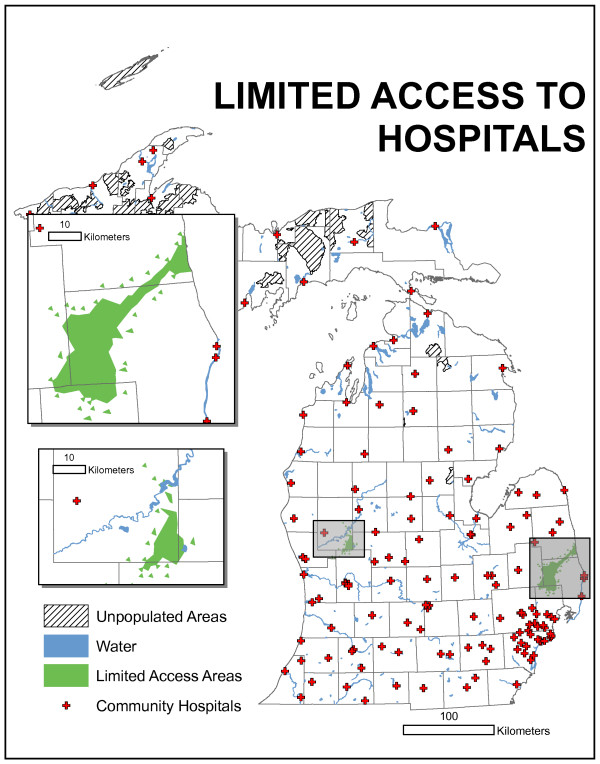
**Results of access-based model using the product present in Figure 6 and the contiguity and population criteria**. Two areas are identified as being "under-served." The upper area is centered north of Grand Rapids and contains four counties, though only a very small portion of Muskegon is actually part of the area. The lower area is north/northeast of Detroit and contains the greater total land area and greater total population of the two regions.

For research purposes, reductions in urban speed were modeled at 50% and 75% but are not presented here. The committee decided to use the "normal" or posted speed limits (Figure 6) for service estimations. Using the product present in Figure 6 and the contiguity and population criteria, in Figure 8, two areas are identified as being "under-served." The upper area is centered north of Grand Rapids and contains four counties, though only a very small portion of Muskegon is actually part of the area. The lower area is north/northeast of Detroit and contains the greater total land area and greater total population of the two regions.

The definition of poorly served as applied here is a contiguous area with a population of at least 50,000 in ZIP codes partially or wholly outside of the 30-minute travel time limit. The limited access region in the eastern Lower Peninsula is the most significantly under-served. Using a conservative measure of contiguity, the under-served population total is 74,450 in year 2000. The region in the western Lower Peninsula also meets the definition of under-served but given the complex spatial pattern requires a more liberal delineation of contiguity. Using the more liberal definition, 61,046 people are under-served.

Both regions contain both partial and complete ZIP codes. It is important to understand that the populations reported are for ZIP code totals. No attempt was made to partition population based on partial ZIP code accessibility. The aggregation of demand by ZIP may result in different types of error. Current and Schilling [22] describe three error sources for aggregated demand allocation problems: A, B, and C. Source A errors consist of either identifying a demand unit as being within the coverage limit when, in fact, it is not, or identifying a demand unit as being outside the coverage when, in fact, it is not. Source B errors are concerned with non-zero distances for demand nodes located coincidentally with facility locations, while source C errors occur when demand is allocated to an incorrect facility due to aggregation. Source B and C errors are not a problem in this model, which does not attempt to allocate demand. Source A errors are a concern. These could result when the centroid of a ZIP code is within 30 minutes of a facility, but a portion of the population within it is not, or when the centroid of a ZIP code is beyond the 30 minute travel time limit, but a portion of the population in the ZIP lies within this limit. We chose the conservative assumption that the entire population of a ZIP was under-served if any part of the ZIP was not within the 30 minute limit. This avoids the first source A error at the cost of a positive bias to both the geographical area and the underserved population. Due to the nature of the application, this was deemed acceptable and preferable to alternative approaches.

Access based models should be used with caution. The primary concern when building an access model is capturing a complete road network. In Michigan, the Department of Transportation records all "M" designated roads, but all private roads, roads managed by the Federal Government, and certain municipal roads are ignored. Capturing a complete road network is particularly problematic with both private drives used by many health care facilities and the extensive private road networks in rural Michigan.

### Optimal hospital locations defined by demand-based location model

**Figure 9:**
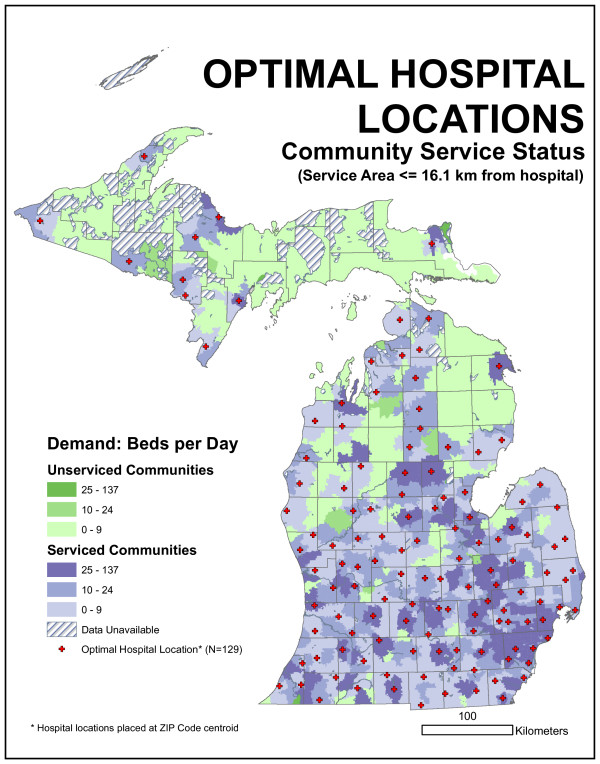
**Spatial representations of the optimal distribution of hospitals for 16.1-kilometer radius distance**. The results of the 16.1-kilometer Maxcover model for the allocation of demand to the existing 129 facilities show over 1,300 more beds per day are filled, indicating a substantial reduction in unmet demand over the existing configuration.

**Figure 10:**
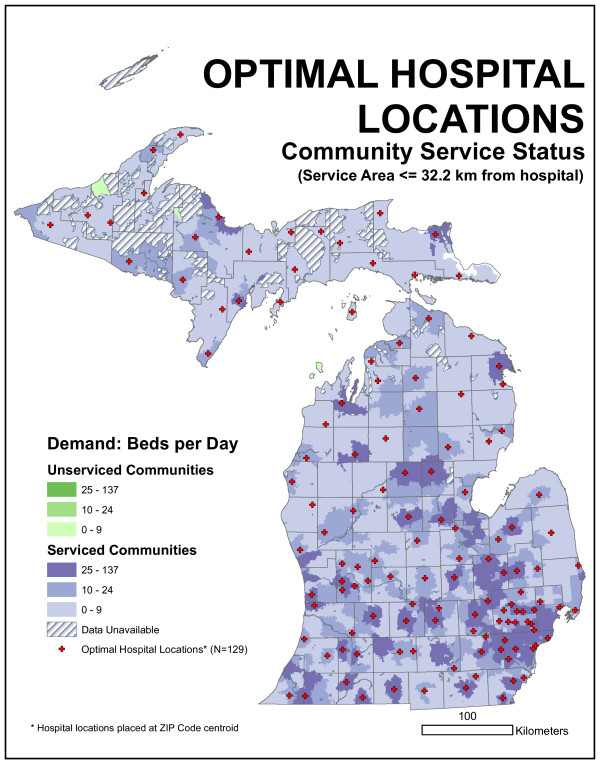
**Spatial representations of the optimal distribution of hospitals for 32.2-kilometer radius distance**. The results of the 32.2-kilometer Maxcover model for the allocation of demand to the existing 129 facilities indicate the 32.2-kilometer optimal model is able to capture all demand in the state. While this sounds impressive, it does not actually represent a substantial improvement over the existing configuration, because most state demand (98.9%) is already within this distance of an existing facility.

Spatial representations of the optimal statistical distribution of hospitals appear in Figures 9 and 10. The 16.1-kilometer (10-mile) optimal model does a clearly better job of capturing statewide demand within the critical radius than the existing distribution. Over 1,300 more beds per day are filled, indicating a substantial reduction in unmet demand over the existing configuration. An inspection of the maps indicates how this reduction occurred. The optimal model placed fewer hospitals in the Detroit area and dispersed hospitals across more rural regions of the state, including the eastern Lower Peninsula, the upper Lower Peninsula, and the western Upper Peninsula. Consequently, the median bed demand per facility actually dropped slightly, while the reduced number of facilities in Detroit handled slightly more people. Nevertheless, there is still a great degree of similarity in the overall pattern; 25 existing facility locations were independently selected as facility locations by the optimal model. Four ZIP code centroids in the Detroit area (Grosse Pointe Park, Birmingham, River Rouge, and Sterling Heights) were assigned more than one thousand beds each. However, average distance from demand points to the closest facility increased over the existing model. Although more people are within 16.1 kilometers of a facility, they were traveling 1.6 kilometers farther on average.

The 32.2-kilometer (20-mile) optimal model was able to capture all demand in the state. While sounding impressive, it did not actually represent a substantial improvement over the existing configuration because most state demand (98.9%) was already within this distance of an existing facility. While optimal from the Maxcover perspective, this solution, like the 16.1-kilometer solution, increased the average distance traveled. Two ZIP code centroids in the Detroit area, Ferndale and Grosse Pointe Park, were assigned more than one thousand beds each. The maps indicate a great degree of similarity with the existing hospital network. Indeed, 53 (of 129) facility locations were identical between the optimal 32.2-kilometer model and the existing network, and 25 of these locations were also in the optimal 16.1-kilometer model.

### Optimal facility locations given existing configuration

**Figure 11:**
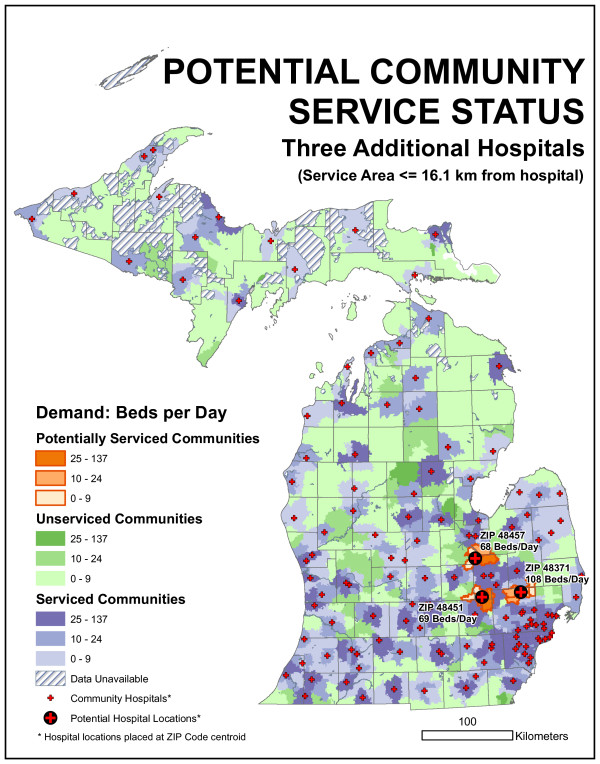
**Results of the demand-based, Maxcover model with a 16.1-kilometer maximum distance**. The three new locations that maximize coverage are 48371, 48451, and 48457. All three locations serve communities on the northern edge of the metropolitan Detroit region.

The "blank slate" results described in the previous section was one location-allocation models implementation. It is also possible to fix sites at the 129 existing locations and identify the optimal 130th, 131st, and 132nd location, given the existing network. This was accomplished using Maxcover models with 16.1 and 32.2-kilometer maximum distances, respectively. While there was no guarantee that a location chosen as optimal in a n-facility model would also be chosen in a n+1 model, that was what happened here (the hospital location chosen for the 130th site was also one of the two chosen in the 131 site model and one of three chosen in the 132 site model). Table 1 provides figures about the facilities chosen, while Figure 11 identifies their locations.

**Table 1:** Optimal sites for 1, 2, and 3 new hospitals, given the existing network

**Model**	**Unmet Demand (beds/day)**	**% Improvement**	**ZIP**	**Facility Size (beds/day)**
**16.1 kilometer existing**	1883.6	-	-	-
**16.1 km. 1st new**	1812.8	3.7%	48371	108
**16.1 km. 2nd**	1744.2	7.4%	48451	69
**16.1 km. 3d**	1676.3	11.0%	48457	68
**32.2 km existing**	159.5	-	-	-
**32.2 km. 1st new**	129.2	19.0%	49632	58
**32.2 km. 2nd**	103.2	35.3%	48619	33
**32.2 km. 3d**	78.5	50.8%	49893	25

The Maxcover model with a 16.1-kilometer maximum distance set to identify 130 locations, with 129 of them 'fixed' to the existing facility ZIP code centroids, the model identified ZIP code 48371 as the best location for the new facility. As Figure 11 shows, this ZIP code is located in northern Oakland County, around the Town of Oxford. As Table 1 shows, unmet demand was reduced by 3.7 percent using the one new hospital with the 129 existing hospitals. The new facility services an average of 108 bed-demand per day. Statewide unmet demand is not actually reduced by 108 beds because part of the supplied demand for the new facility had been served by existing hospitals.

When the same model is run to identify 131 locations, with 129 of them 'fixed' to the existing facility ZIP code centroids, the model identifies two ZIP codes, 48371 and 48451, as the best locations for the two new facilities. 48451 is the ZIP code for Linden, in southern Genesee County. Combined, these hospitals reduce unmet demand by 7.4 percent. The Linden location serves 69 beds per day of demand. Finally, running the same model to find 132 locations, with 129 of them fixed, the three new locations that maximize coverage are 48371, 48451, and 48457. The 48457 ZIP code serves the Town of Montrose in northwestern Genesee County. This new facility serves 68 beds per day of demand, and these 132 facilities handle 1676.3 demand per day, an 11 percent improvement over the existing 129 Michigan community hospitals. As Figure 11 shows, all three locations serve communities on the northern edge of the metropolitan Detroit region.

**Figure 12:**
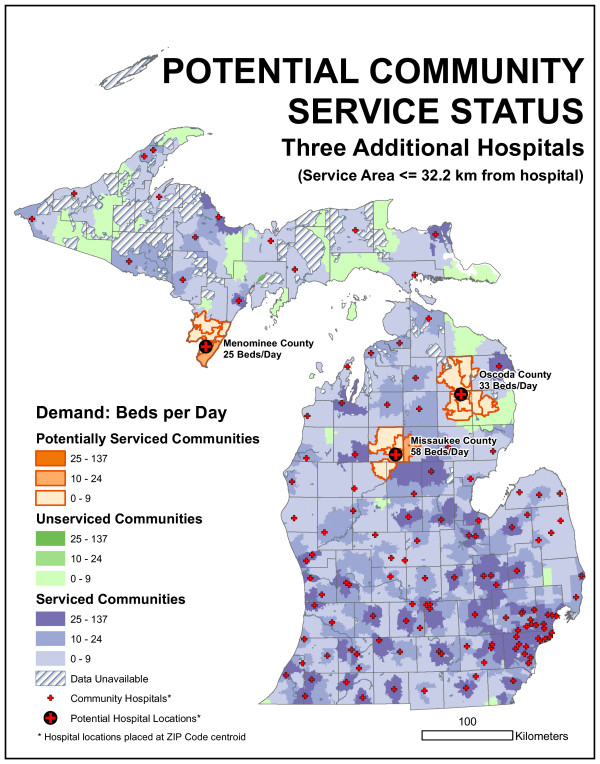
**Results of the demand-based, Maxcover model with a 32.2-kilometer maximum distance**. The three new locations that maximize coverage are 49632, near Falmouth in southeastern Missaukee County, 48619, serving the Village of Comins in northeastern Oscoda County, and 49893, serving the Town of Wallace in southern Menominee County near the Wisconsin border.

A very different set of solutions arises when the model is run with a 32.2-kilometer maximum distance. Figure 12 illustrates the facility locations using this model. For a single new facility, the model selects ZIP code 49632, near Falmouth in southeastern Missaukee County. This facility would serve 58 beds per day of demand and would improve the existing 32.2 km model by 11%. For two new facilities, the model also identifies 48619, serving the Village of Comins in northeastern Oscoda County. This site serves 33 beds per day and, along with the other facilities, reduces statewide unmet demand to 103.2 beds per day, an improvement of 35.3% over the existing hospitals. If three new sites are chosen, the third choice is 49893, serving the Town of Wallace in southern Menominee County near the Wisconsin border. This facility would serve 25 beds per day of nearby demand. The final site is an interesting example of boundary effects in spatial analysis. The nearby city of Menominee is a regional center but does not have a hospital; in fact, the closest hospital is just across the state line in Marinette, Wisconsin. However, hospitals in bordering states are not included in the Michigan data set; similar issues may affect demand and allocation along the Ohio and Indiana borders as well.

We performed this analysis using ZIP code centroids as proxies for the complex distribution of patients and hospital locations. This was primarily to ensure that the focus would remain on regional rather than site specific scales of analysis. The use of patient days data for a specific year provides a useful snapshot of the spatial distribution of demand at a particular moment. However, we are unable to quantify variability in occupancy either at sub-annual or inter-annual periods. We must assume that 2002 is a representative year, and that bed occupancy is roughly uniform throughout the year. More significantly, perhaps, these data alone cannot account for longer-term demographic and technological trends that could substantially impact the geography of bed supply and demand. Just as the current distribution of community hospital facilities is a product of Michigan's past, so will these results become a relic of the situation in 2002 for a future generation.

There are four main concerns related to this type of modeling. First, the employment of ZIP code centroids for both facility location and demand origin and the use of straight-line distance are simplifications of the actual geography and are potentially significant sources of error. Spatial aggregation of demand by ZIP was not a modeling choice but instead was inherent in the data available for this study; this made disaggregated analysis impossible. Unlike the access-based model, this model was sensitive to aggregation error from all three sources described in Current & Schilling [22] and previously in this paper. Therefore, an approach that could estimate the total error due to aggregation was adopted from Francis et al. [23]. This work identified a very strong negative relationship between objective function error magnitude and the ratio of demand nodes (q) to sites (p). As q/p dropped below 50, relative error increased sharply above one percent. At q/p of approximately 1, error was between five and six percent. In the present study, this ratio would correspond to 893/~130, or 6.9, which corresponds to a high relative error rate and an unknown impact on the location of model-selected facilities.

With respect to distance, euclidean distance provides a useful first cut, but on Michigan's township-range rural road system, road distances could be to be up to twice as long. Network distance might therefore be preferable, and modeled travel time superior to that. Second, patients choose hospitals for diverse reasons; spatial proximity is only one. For example, the geography of the referral network may be of particular relevance. Additionally, the importance of proximity in the delivery of health care services is variable. Third, the outcome of these models is highly dependent on the selection of model form and of key input parameters. A P-median model optimal result is different from a Maxcover optimum. The results shown here suggest that changing the maximum coverage radius from 16.1 to 32.2 kilometers changes the proportion of population covered, the optimal configuration of hospitals, or the identification of an optimum location for the 130th community hospital facility. Fourth, the software implementation used here cannot employ facility capacity information in the location model. This means that the model assumes that a facility can handle any amount of demand, when in fact hospitals are constrained by their number of beds. Although the results presented here suggests that this is not a serious problem for Michigan, which experiences average bed occupancy rates well below capacity, it would be preferable to use a system that can account for facility capacity.

## Conclusion

Michigan is clearly a state in transition. While the population as a whole is less dynamic than other states in the country, there are significant spatial and demographic transitions occurring within its boundaries. Implications of this changing demographic landscape are considerable for public health policy; the process of selecting locations for new hospitals has been and continues to be rife with political activity if only for the significant financial investments and potential rewards involved. We hope that the results presented here are useful, but they should be viewed with some caution. It is important to be clear about the limitations of the models employed in this work. There are always gaps between data and the phenomena they represent and between process models and the complex real world that they attempt to emulate.

The access-based model identified two specific under-served areas with populations greater than 50,000 outside 30 minutes travel time, while maintaining contiguity constraints. These areas were ultimately considered too small to warrant new facilities now, but will be monitored for future population changes as both areas are rapidly growing bedroom communities. The large rural underserved populations in the northern Lower Peninsula and in the Upper Peninsula simply do not meet economic constraints, and no new health care facilities have been proposed in these regions by the private sector.

The demand-based location-allocation models were intended to accomplish several objectives:

• Foster a discussion on the meaning of optimal locations and to highlight the sometimes large differences between alternative models and their solutions

• Highlight important geographical characteristics of the existing demand and supply of licensed community hospital beds across the state

• Quantify the presence of bed demand – using actual data – distant from currently existing facilities

• Identify optimal locations for new hospitals, based on narrowly defined sets of criteria

We were able to quantify the quality of the spatial coverage provided by Michigan's existing distribution of community hospitals. Table 2 indicates that, using certain location models, the existing configuration is suboptimal but possesses desirable qualities, such as relatively short distances from most demand points to the nearest facility. In addition, many locales with existing facilities were also identified as optimal locations by the models. Second, we demonstrated that choice of key model parameters, such as the maximum distance, has a profound effect on the allocation of facilities. Although these models were comparable in some ways, they also showed substantial differences. Third, the identification of demand regions that are distant from existing facilities is a direct function of the maximum distance parameter. Consequently, the optimal placement of new facilities, given the 129 ZIP codes at which hospitals are currently situated, is profoundly affected by the distance parameter. In the analysis presented here, optimal locations in different model runs were situated in entirely different parts of Michigan due to a 16.1-kilometer change in the maximum distance.

**Table 2:** Comparison of key measures for different hospital demand allocation models

**Model**	**Unmet Demand (beds (%))**	**Avg Distance (kilometers)**	**Median Facility Demand (beds)**	**Maximum Facility Demand (beds)**
**16.1 kilometer existing**	1883.6 (12.7%)	7.6	46.1	782.1
**16.1 kilometer optimal**	509.9 (3.4%)	9.2	38.7	1245.5
**32.2 kilometer existing**	159.5 (1.1%)	12.7	62.7	782.1
**32.2 kilometer optimal**	0 (0%)	13.4	57.3	1397.4

Ultimately this paper is concerned with the intersection of spatial analysis and policymaking. It is representative of the kind of work that can be accomplished in a data-rich environment with substantial computational resources and a fruitful engagement between research scientists and public health professionals. Using best scientific practice to identify locations of under-served populations based on many factors provides policymakers with a powerful tool for making good decisions.

## Methods

### Access-based model: a raster model of travel time

Identifying travel time is a widely recognized application of modern consumer GIS systems, like OnStar™ and handheld GPS mapping systems. These tools rely on assumptions of locations and travel along a network and thus are entirely restricted to the publicly defined road network. This assumes that all travel begins on a road or on the network. Like cell phone coverage, the road network leaves significant gaps in statewide coverage maps. Further, these gaps may in many cases comprise areas with a) road networks too new to be counted in the public system; b) areas of undocumented private or national road designations; or c) urban districts with significant industrial facilities. Consequently, a raster grid based model that accounts for all places was proposed. The grid model required more computational power and storage than the network model, but provided a complete spatial representation of state hospital and health coverage and avoided unrealistic degrees of precision. Identifying an appropriate spatial resolution for the raster involved substantial experimentation; the final statewide model employed 1-kilometer cells. The final raster model was comprised of cell values indicating the approximate travel time to the nearest community hospital for locations in that cell. This final product required the development of intermediate raster models representing the cost, in minutes, to traverse each cell.

The road network used is publicly available from the Michigan Center for Geographic Information. The geographical positions and density of roads are augmented by attribute data for each road segment. Segment codes are derived from the Michigan Department of Transportation (MDOT) functional class of road designations. This class system uses the United States Department of Transportation's National Functional Classification (NFC) system. There are three major types (Arterial, Collector, and Local) within this system; roads are further divided into urban and rural categories (Table 3). These roads are officially "M" designated roads.

**Table 3:** MDOT National Functional Classification (NFC) code road classes

1 – Rural Interstate (principal arterial)	11 – Urban Interstate (principal arterial)
2 – Rural Other Principal Arterial (non-freeway)	12 – Urban Other Freeway (principal arterial)
5 – Rural Other Freeway (principal arterial)	14 – Urban Other Principal Arterial (non-freeway)
6 – Rural Minor Arterial	16 – Urban Minor Arterial
7 – Rural Major Collector	17 – Urban Collector
8 – Rural Minor Collector	19 – Urban Local
9 – Rural Local	0 or uncoded – not a certified public road

Speed limits are defined by road type, and, in Michigan, range from 25 to 70 miles per hour (40.2 to 112.7 kilometers per hour). No central organization manages or records speed limit information statewide. MDOT records speed limit information for M designated roads only. Thus, speed limits for representative road types were based on the speed limits of representative roads in the Mid-Michigan area. National guidelines for speed limit determination state that speed limits be based on the 85th percentile speed of all travelers over any given road segment. Thus, roads change speed limits over their entire length but should do so within a 10 mph range (16.1 kph) or be redefined into another functional class.

### Calculating traversal costs

To produce maps and other data products displaying specific times, ESRI Arc/Info GRID based spatial analysis tools were employed. There are two existing classes of functions that can be used. The simplest class is the basic Euclidean distance function class, which have been used to create buffers or boundaries around a site, hospital, of some specified distance. These functions have a long history in applied geographic research; however, they fail to effectively capture the variations in landscape and, most importantly for this project, transportation networks. Thus, weighted distance functions were tested and, ultimately, PATHDISTANCE was selected for the travel time methodology. These classes of functions are similar to Euclidean distance functions, but instead of calculating the actual distance from one point to another, they determine the shortest weighted distance (or accumulated travel cost) from each cell to the nearest cell in the set of source cells. A second exception is that weighted distance functions apply distance not in simple distance measures but in cost units. The term "cost" is the precise and correct term, but may be viewed very specifically for this research as "time."

All weighted distance functions require a source grid and a cost grid. A source grid indicates starting locations for calculating total cost. In this analysis, vector point data representing the positions of existing community hospitals were employed as the source grid. A cost grid depicts the cost, in effort or time, involved in moving through any particular cell. The value of each cell in the cost grid is assumed to represent the cost-per-unit distance of passing through the cell, where a unit distance corresponds to the cell dimensions. For this project, costs indicate the time required to traverse a cell based on the slowest speed limit of any road within that 1 km cell. This conservative estimate appeared desirable given the risks of underestimating actual travel time to the nearest hospital.

The cost values assigned to each cell are per-unit distance measures for the cell. That is, if the cell size is expressed in meters, the cost assigned to the cell is the cost necessary to travel one meter within the cell. If the resolution is 1000 meters, the total cost to travel either horizontally or vertically through the cell would be the cost assigned to the cell times the resolution (total cost = cost * 1000). To travel diagonally through the cell, the total cost would be 1.414214 times the cost of the cell times the cell resolution (total diagonal cost = 1.414214 [cost * 1000]). By interpreting the costs stored at each cell as the cost-per-unit distance of travel through the cell, the analysis becomes resolution independent.

The PATHDISTANCE function creates an output grid in which each cell is assigned the accumulative cost from the lowest cost source cell. The PATHDISTANCE function then determines the minimum accumulative-travel cost from a source to each cell location on a grid. PATHDISTANCE not only calculates the accumulative cost over a cost surface, it also does so while compensating for the actual surface distance that must be traveled and for the horizontal and vertical factors influencing the total cost of moving from one location to another.

The specific output product was the total accumulative cost-distance grid. This grid stored the least-cost-accumulated distance for each cell that resulted from the least costly source cell. The least-cost-accumulated distance grid was transformed into a map product. The map product was used in a traditional map algebra process "overlay" with a ZIP code map containing year 2000 census data. The final output products of this process were two-fold: a ZIP code database that identifies unique ZIP codes and fractions of ZIP codes including multiple fractions of the same ZIP code, all outside the 30 minute travel time boundary. There were both map and database products. The Results section, above, presents the output map products.

### Demand-based model: hospital and patient data

The committee supplied us with two crucial data sets for developing this analysis: Michigan's community hospitals and state ZIP codes with associated patient days. The first of these was a list of 139 community hospitals with addresses and licensed bed capacity for 2002. A brief review of these data reveals some interesting characteristics about the number and variability of capacity:

• 24,924 beds statewide

• Greatest bed capacity: 903(Henry Ford – Detroit)

• Smallest bed capacity: 8 (Paul Oliver-Frankfort)

• Capacity Statistics: Mean: 179 beds; Median: 106 beds; Std Dev: 174 beds

Half of the state's community hospitals have fewer than 106 beds. The inner quartile range, indicating the middle 50%, lies between 53 and 269 beds. Several very large facilities with hundreds of licensed beds are far above this inner quartile range. Because fine spatial precision was not deemed necessary or desirable for this portion of the research, each hospital's position was identified simply as the central point (centroid) of the ZIP code. The goal is to identify hospital demand at a regional level, not to identify site-specific locations for facilities. These hospitals are located in 129 different ZIP codes. One code – 48201 in Detroit – contains 5 facilities with a total of 1,809 licensed beds. For this component of the project, we will consider Michigan as having 129 locations at which hospital beds are available; these locations may include more than one facility. Most facilities, and most licensed beds, are in densely populated southeastern Michigan. A regularly spaced pattern of hospital facilities characterizes most of the northern, rural parts of the state.

The second data set was a list of 907 ZIP codes across Michigan with their associated aggregate patient days for 2002. Only patient days at community hospitals were included. We were able to find the spatial location for 893 of those ZIP code centroids. Looking at the statistical distribution of the patient day data reveals some important characteristics:

• Total number: 5,407,985 patient days

• Fewest Patient Days: 18 (48824 – East Lansing, MSU Campus)

• Greatest Patient Days: 49,506 (48180 – Taylor)

• Patient Days by ZIP: Mean: 6,055; Median: 2,533; Std Dev: 8,206

• The inner quartile range (middle 50%) of this data range from 996 to 7,654.

Some of the smallest numbers in the data set represent special cases. 48824 is the Michigan State University campus ZIP code. The next smallest, with 19 days, is the ZIP code for Detroit Metro Airport. Other university campus ZIP codes feature prominently at the bottom end of the patient day rankings. It is likely that many residential students requiring hospitalization report their parents' home address, thereby making interpretation of the values difficult. Patient days are not an ideal variable for this analysis, which is concerned with occupancy rates. Dividing patient days in each ZIP code by 365 provides a figure representing average daily demand from each ZIP code. The statewide daily average bed demand is 14,817. This figure can be compared to the total supply of 2002 licensed beds by community hospitals to calculate a statewide average daily occupancy rate of 59.4%. Of course, this average is only an approximation of any particular daily rate. We do not have access to data that would enable us to identify the variation about that average.

**Figure 13:**
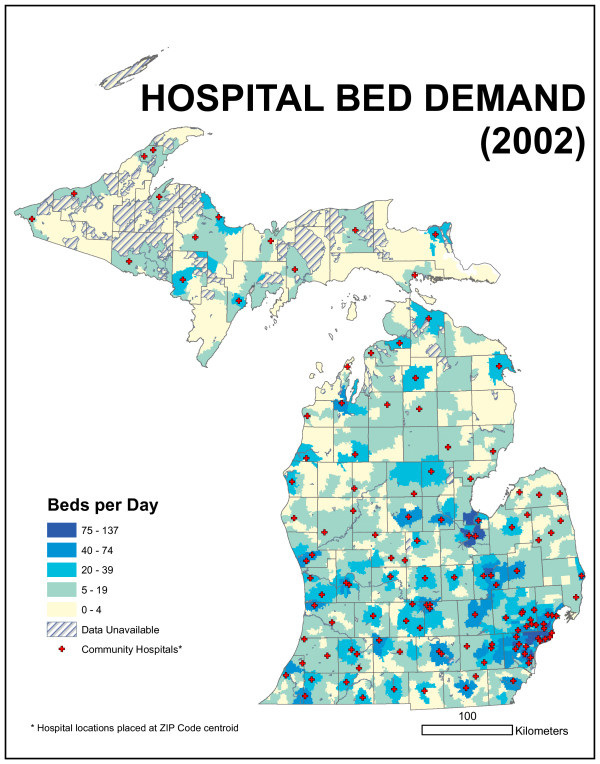
**Map of bed demand per day**. High values are located around metropolitan Detroit, Grand Rapids, and other urban population centers. Lower values are located in rural parts of the state.

We would expect bed demand per day to vary geographically across the state and to generally follow the spatial distribution of population. Figure 13 is a map of bed demand per day. High values are located around metropolitan Detroit, Grand Rapids, and other urban population centers. Lower values are located in rural parts of the state. ZIP code size varies by more than an order of magnitude in Michigan; they are larger in areas of low population density and smaller in high-density areas. This means that rural ZIP codes can still include substantial populations, simply because they occupy so much area.

### Demand locations distant from existing hospital locations

**Figure 14:**
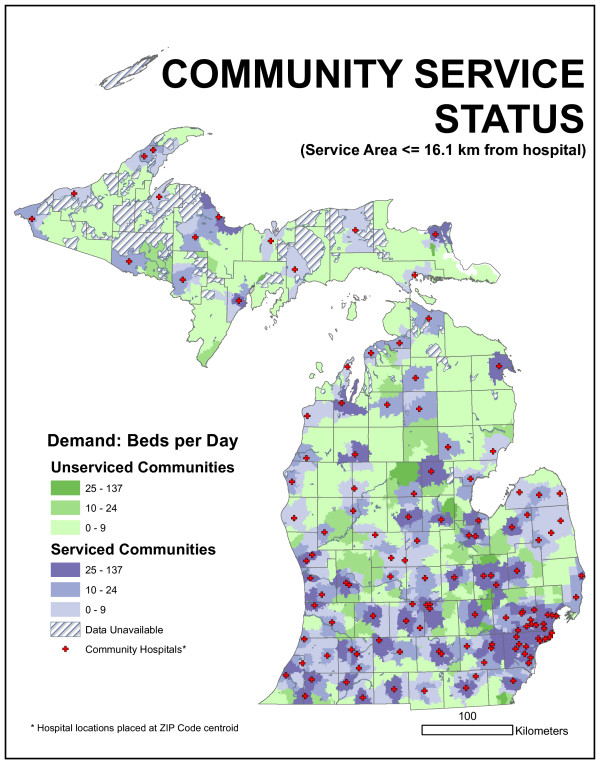
**Map of community service status for 16.1-kilometer radius distance**. 1,887 beds per day were demanded by ZIP centroids more than 16.1 kilometers from the nearest existing hospital facility. This represents 12.7% of the average statewide daily bed demand.

**Figure 15:**
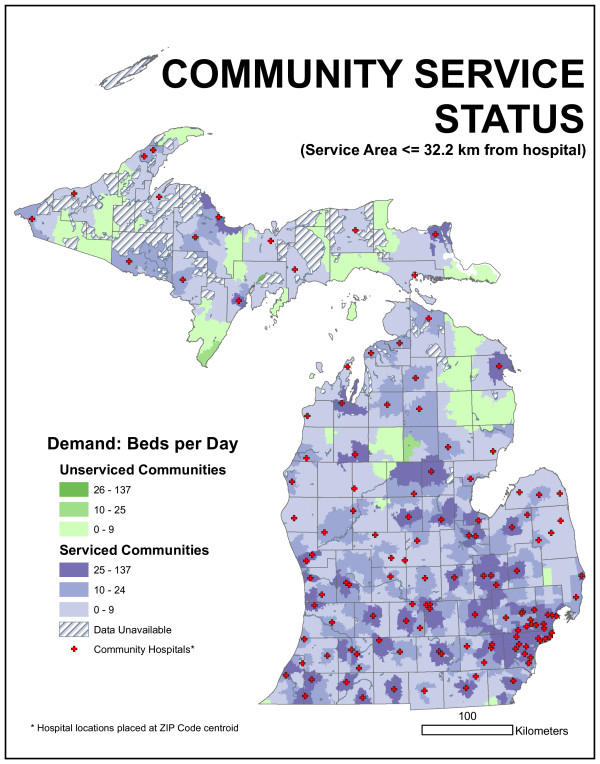
**Map of community service status for 32.2-kilometer radius distance**. Unmet demand drops to 160 beds per day (1.1%) when 32.2-kilometer radius is employed.

We could evaluate the existing facility arrangement by allocating daily bed demand in each ZIP code to the closest facility up to any particular distance. The committee suggested 16.1 km (10 mile) and 32.2 km (20 mile) radii. This distance was not based on network distance but on Euclidean distance. ZIP code locations that fell outside this distance represented sources of unmet demand. Arc 8.2 and custom programming were used to quantify this demand. We found 1,887 beds per day were demanded by ZIP centroids more than 16.1 kilometers from the nearest existing hospital facility. This represents 12.7% of the average statewide daily bed demand. If a 32.2-kilometer radius is employed, unmet demand drops to 160 beds per day (1.1%). Figures 14 and 15 characterize this unmet demand for both distances for the entire state.

It may be useful to consider the amount of demand allocated to each facility using this simplistic distance-based model. Allocated demand represents the average number of occupied beds for each ZIP code with a facility. Beds utilized per day for the 129 facilities under the 16.1-kilometer model range from 5.6 to 782 with a median bed demand of 46.1 and an inner quartile range (middle 50%) from 20.5 to 140.5 bed utilization. The average distance from a served demand point to its nearest facility was 7.6 kilometers. For the 32.2-kilometer model, average daily bed occupancy ranges from 8.4 to 782 with a median of 62.7 and an inner quartile range of 36.3 – 162.9. Served demand points were an average of 12.7 kilometers from their nearest facility. These higher utilization rates and distances (compared to the 16.1 kilometer radius model) were a direct function of the larger demand covered by the 32.2-kilometer radius. Neither model accounted for actual facility size, but numbers appeared reasonable. As an example, note that the maximum occupancy, 782, was the same facility for both 16.1 and 32.2 kilometer models. This was for ZIP code 48202, which is the location of Henry Ford, the largest hospital in the state with 903 licensed beds.

### Optimal spatial distribution

We have determined an optimal spatial distribution of community hospital facilities would situate facilities so as much bed demand as possible was within a given distance of the nearest facility, an example of a maximal covering location model. Executing this model required that the number of facilities to position be known in advance, along with the covering distance. The number of facilities was set at 129 (the number of unique ZIP codes with existing facilities). The committee indicated that interesting distances to consider would be 16.1 and 32.2 kilometers. While the model environment can employ road network distance, the present analysis uses Euclidean distance as a rough proxy for travel time.

Analyses were conducted in Arc 8.2 using the parameters just indicated and the data described in the previous section. Table 2 describes the results of the 16.1 and 32.2-kilometer Maxcover models, as well as comparable statistics for the allocation of demand to the existing 129 facilities. Spatial representations of these optimal distributions of hospitals appear in the Results section, above.

The GIS implementation we employed was unable to incorporate capacity restraints in location models. This means that we assumed that any facility could handle any amount of demand. It was certainly possible that unrealistic amounts of demand could be assigned to individual facilities. The last few columns in Table 2 may be compared with the actual statistics on the distribution of licensed beds at the beginning of the Data Section above to consider this problem. Median demand was substantially lower than actual median bed capacity (106 beds) for the 16.1-kilometer models. While median bed demand is also less for the 32.2-kilometer model, it was not substantially different than the median hospital capacity multiplied by the average occupancy rate (0.594), or 63 beds. That is, on an average day in 2002, an average hospital has patients in 63 beds. Maximum bed demand values were also "in the ballpark": the actual maximum number of licensed beds in any ZIP code is 1,809. This number, when multiplied by the average occupancy rate, was comparable to the maximum demanded by the optimal location models.

## Competing interests

The author(s) declare that they have no competing interests.

## Authors' contributions

JPM and AMS both contributed to all aspects of the work: manuscript writing, data development, question formulation, literature review and modelling. REG contributed to manuscript writing, demographic analyses, and cartography. PV contributed to manuscript writing, data development and access modelling. MJF contributed to manuscript writing, database development, and cartography.
